# Medical and Financial Consequences of Using PCSK9 Inhibitors for Managing Hypercholesterolemia in Saudi Arabia: A Historical Cohort Study

**DOI:** 10.3390/healthcare13192428

**Published:** 2025-09-25

**Authors:** Yazed AlRuthia, Khlood Khaled Almutairi, Norah Abdulaziz Aljammaz, Aseel Alsuwayegh, Miteb A. Alanazi, Rasha Fahad AlSulaiman, Tareq Majed Alfaori, Numan Alabdan

**Affiliations:** 1 Department of Clinical Pharmacy, College of Pharmacy, King Saud University, Riyadh 11451, Saudi Arabia; 2 Pharmacoeconomics Research Unit, College of Pharmacy, King Saud University, Riyadh 11451, Saudi Arabia; khloodkhaled.a@gmail.com (K.K.A.); aljammazna@gmail.com (N.A.A.); 3 Corporate Department of Pharmacy, King Saud University Medical City, Riyadh 12372, Saudi Arabia; aalsuwayegh@ksu.edu.sa (A.A.); mitalanazi@ksu.edu.sa (M.A.A.); 4 Pharmacy Service Administration, King Fahad Cardiac Center, King Saud University Medical City, Riyadh 11451, Saudi Arabia; rbinsulaiman.c@ksu.edu.sa; 5 Dr. Sulaiman Al Habib Medical Group, Inpatient Pharmacy, Al Khobar 34423, Saudi Arabia; tareq.alfaori@drsulaimanalhabib.com; 6 Pharmaceutical Care Department, King Abdulaziz Medical City, Ministry of National Guard Health Affairs, Riyadh 14611, Saudi Arabia; abdann@mngha.med.sa

**Keywords:** hypercholesterolemia, PCSK9 inhibitors, evolocumab, alirocumab, hospitalization, cost-effectiveness analysis, health expenditures

## Abstract

**Background:** Managing hypercholesterolemia is essential for reducing health risks and costs. Proprotein Convertase Subtilisin/Kexin type 9 (PCSK9) inhibitors are recommended for patients with high low-density lipoprotein cholesterol (LDL-C) levels at risk for cardiovascular disease, especially those on maximum doses of statins and ezetimibe. However, their cost-effectiveness is unclear, particularly in Saudi Arabia, where cardiovascular disease is prevalent. The main objective of this study was to evaluate the costs and outcomes of PCSK9 inhibitors versus statins and ezetimibe. **Methods:** A multicenter retrospective study reviewed charts of adults (≥18 years) with hypercholesterolemia treated with PCSK9 inhibitors (evolocumab or alirocumab) for at least 12 months. Outcomes included LDL-C reduction and cardiovascular-related hospitalizations, with direct medical costs estimated via micro-costing and adjusted for confounders. **Results:** The analysis included 118 patients on PCSK9 inhibitors and 304 on statins plus ezetimibe. Mean LDL-C reductions were 1.432 mmol/L [95% CI: 0.964 to 1.899] for PCSK9 inhibitors and 0.644 mmol/L [95% CI: 0.464 to 0.823] for the other group. Cardiovascular-related hospitalizations averaged 0.645 for PCSK9 inhibitors compared to 0.808 for statins plus ezetimibe. The annual cost for PCSK9 inhibitors ranged from USD 4024 [95% CI: 3786.80 to 7947.91] to USD 7559 [95% CI: 7331.35 to 11,509.66]. In 99.13% and 98.78% of bootstrap distributions, PCSK9 inhibitors led to greater LDL-C reductions and fewer hospitalizations. **Conclusions:** The use of PCSK9 inhibitors for managing hypercholesterolemia was associated with a greater reduction in LDL-C levels and fewer cardiovascular-related hospitalizations. However, the more modest LDL-C reduction compared to clinical trials, combined with the high acquisition cost of PCSK9 inhibitors, underscores the need to provide significant price reductions to improve patient access to these lipid-lowering agents.

## 1. Introduction

Cardiovascular diseases (CVDs) are the leading cause of death worldwide, responsible for approximately 17.9 million fatalities each year, which accounts for 32% of all global deaths [1]. Most of these deaths are due to heart attacks and strokes, with a significant number occurring in low- and middle-income countries [1,2,3]. In 2021, CVDs were estimated to have caused 20.5 million deaths [2,3]. Patients with hypercholesterolemia, characterized by low-density lipoprotein cholesterol (LDL-C) levels higher than 4.9 mmol/L for individuals without cardiovascular disease (CVD), are at an increased risk for major adverse cardiovascular events (MACE) (nonfatal stroke, nonfatal myocardial infarction, and cardiovascular death) [2,4,5].

In Saudi Arabia, CVDs pose a significant public health challenge, primarily due to high levels of LDL cholesterol and other associated risk factors [6]. The most common forms of CVD in the Kingdom are ischemic heart disease and cerebrovascular disease [7]. More than 30% of adults in Saudi Arabia are at risk for CVD-related events, with dyslipidemia characterized by high LDL and low high-density lipoprotein (HDL) cholesterol affecting 68.6% of the population [6,7]. It is projected that the economic burden of CVDs will triple by 2035 [8,9].

The management of hypercholesterolemia is multifaceted and involves a comprehensive approach that encompasses diet, exercise, and pharmacological therapies [10]. Statins, HMG-CoA reductase inhibitors, have long been the mainstay of therapy for hypercholesterolemia. They have proven effective in reducing the risk of CVD due to their effects on lowering LDL-C levels [10,11]. Another commonly prescribed medication, often used in conjunction with statins, is ezetimibe, which has also been shown to be effective in reducing LDL-C levels [10,12]. However, these medications often fall short of the recommended 50% reduction in LDL-C levels, as advised by the American Heart Association/American College of Cardiology for patients with chronic coronary disease (CCD) or Familial Hypercholesterolemia (FH) [10,12]. This limitation underscores the need for newer therapeutic agents, such as the Proprotein Convertase Subtilisin/Kexin type 9 (PCSK9) Inhibitors (e.g., evolocumab, alirocumab), small interfering RNA (siRNA) treatment (e.g., inclisiran), and angiopoietin-like 3 (ANGPTL3) (e.g., Evinacumab) for patients who did not achieve their target LDL-C levels despite being on maximally tolerated statins and ezetimibe [10,13,14,15].

PCSK9 inhibitors have emerged as the most frequently used lipid-lowering agents for patients who do not meet their target low-density lipoprotein cholesterol (LDL-C) levels while being treated with the maximum tolerated dosages of statins combined with ezetimibe, as recommended by guidelines set forth by the American Heart Association (AHA) and the American College of Cardiology (ACC) [10,16]. These inhibitors, which play a crucial role in cholesterol management, are primarily indicated for primary prevention in patients with familial hypercholesterolemia (FH). However, their use extends to secondary prevention in individuals with cardiovascular disease (CVD), regardless of the presence of other risk factors such as diabetes, hypertension, or chronic kidney disease. Such indications are supported by the recommendations found within the guidelines of the AHA/ACC, the European Society of Cardiology, and the Saudi Guidelines for the Management of Dyslipidemia [10,17,18,19].

The rationale for the integration of PCSK9 inhibitors into treatment regimens for hypercholesterolemia can be attributed to their enhanced efficacy in reducing LDL-C levels compared to conventional lipid-lowering therapies. Standard treatments, including statins, bile acid sequestrants, and ezetimibe, have been the target of extensive clinical investigations, demonstrating that PCSK9 inhibitors, such as evolocumab and alirocumab, provide superior results in cholesterol reduction [20,21].

While randomized controlled trials have elucidated that the numbers needed to treat (NNT) for risk reduction in major adverse cardiovascular events (MACE), strokes, and the necessity for coronary revascularization are more favorable for patients receiving evolocumab in comparison to placebo, a direct head-to-head comparison with alirocumab has not been conducted [22]. In a systematic review evaluating the available evidence, it was found that alirocumab exhibited stronger evidence for reducing cardiovascular events and mortality risk in high-risk CVD patients who had not achieved their LDL-C targets. Alternatively, evolocumab demonstrated greater efficacy among patients with heterogeneous FH. However, it is critical to note that the existing studies did not definitively establish which of the two PCSK9 inhibitors has superior benefits due to methodological limitations in analyzing cardiovascular endpoints [23]. Additionally, while clinical trials have shown that PCSK9 inhibitors can lead to a reduction of 50% or more in LDL-C levels, real-world observational studies have not confirmed these findings. Although these studies have demonstrated significant reductions in LDL-C and total cholesterol levels, the magnitude of reduction was not comparable to that observed in clinical trials. This discrepancy may be attributed to variations in patient characteristics and adherence levels [24,25].

In addition to concerns regarding clinical effectiveness, the cost-effectiveness of these lipid-lowering agents presents another layer of complexity due to their high acquisition costs and the lack of robust real-world evidence validating their economic viability among patients with heterozygous FH or those at heightened risk for atherosclerotic cardiovascular disease (ASCVD) [26,27]. Although various published cost-effectiveness analyses conducted across different jurisdictions indicate that PCSK9 inhibitors outperform standard care for patients with FH or ASCVD, and for individuals intolerant to statins, these studies have predominantly relied on simulation models generated from data obtained in landmark clinical trials. Notably, they also suggest that significant reductions in the annual costs of evolocumab or alirocumab (ranging from 20% to 88%) would be necessary for these treatments to be considered cost-effective [28,29,30,31,32].

A specific cost-effectiveness analysis conducted in Saudi Arabia from the standpoint of public healthcare payers found that the use of evolocumab is cost-effective for patients with clinically significant ASCVD presenting with baseline LDL-C levels of ≥70 or ≥100 mg/dL, as well as for those with heterozygous FH, when applying a cost-effectiveness threshold of up to three times the gross domestic product (GDP) per capita [33]. However, under newly released cost-effectiveness thresholds for Saudi Arabia, estimated between USD 13,333 and USD 20,000 per quality-adjusted life year (QALY), evolocumab would only demonstrate cost-effectiveness when administered to individuals with heterozygous FH [34].

Given these insights, the objective of this study was to conduct real-world cost-consequence analysis to provide valuable information to healthcare decision-makers in Saudi Arabia concerning the medical impact and financial implications of incorporating PCSK9 inhibitors into the treatment strategy for patients diagnosed with heterozygous FH, ASCVD, and those at elevated risk for CVD, especially in comparison to traditional treatments such as statins and ezetimibe.

## 2. Methods

### 2.1. Study Design and Data Source

This study was a historical cohort analysis involving patients with baseline LDL-C levels of 1.8 mmol/L (70 mg/dL) or higher, or 2.6 mmol/L (100 mg/dL) or higher. The participants either had atherosclerotic cardiovascular disease (ASCVD) or were at high risk for ASCVD, such as individuals with heterozygous FH. They were treated with either PCSK9 inhibitors (e.g., evolocumab or alirocumab) or statins (e.g., simvastatin or atorvastatin) in combination with ezetimibe. The patients were retrospectively recruited from the electronic medical records (EMRs) of three tertiary care hospitals.

Adult patients aged 18 years and older treated with PCSK9 inhibitors for at least 12 months were matched with a cohort of patients receiving statins plus ezetimibe. Patients with malignancies and those who had a follow-up period of less than 12 months were excluded from the study. Data collected from the EMRs included demographics such as age and gender, body mass index, duration of illness and treatment, and other chronic health conditions (such as chronic kidney disease, diabetes, hypertension, myocardial infarction, and stroke). Lipid profile measures (including LDL-C, HDL-C, triglycerides, and total cholesterol), the frequency of cardiovascular-related hospitalizations (such as heart failure, myocardial infarction, stroke, aortic aneurysm, and arrhythmias), and the lengths of hospital stays were also recorded. Healthcare cost data were sourced from the Saudi Ministry of Health’s Cost Center, while prescription drug prices were obtained from the Saudi Food and Drug Authority’s (SFDA) website. No adjustments for inflation were made since forecasting was not conducted in the current study.

### 2.2. Study Outcomes and Data Analysis

The primary clinical outcomes of this study were the mean difference in LDL-C levels at baseline and follow-up, and the mean number of cardiovascular-related hospitalizations (including heart failure, myocardial infarction, stroke, aortic aneurysm, and arrhythmias) as documented in the patients’ EMRs. Micro-costing was conducted to estimate the direct medical costs of treating patients with hypercholesterolemia, which included laboratory tests, imaging studies, outpatient visits, prescription medications, emergency department visits, hospitalizations, and all other health services provided [35]. A paired *t*-test was used to examine the mean difference in LDL cholesterol, HDL cholesterol, and total cholesterol between patients at baseline and follow-up. Multiple covariate-adjusted linear mixed-effects models were conducted to generate the adjusted mean difference for the lipid panel measures and cardiovascular-related hospitalization, controlling for different covariates (e.g., comorbidities, age, gender, disease and treatment durations, and follow-up periods). Inverse probability of treatment weighting in which propensity scores based on the patients’ characteristics (age, gender, durations of illness and treatment, follow up periods, and comorbidities) were generated and then weights were assigned using the inverse propensity scores to adjust for confounders among the patients treated with PCSK9 inhibitors (e.g., evolocumab, alirocumab) and their counterparts treated with statins and ezetimibe. Non-parametric bootstrapping with 10,000 replications was performed at a statistical significance level of *p* = 0.05 to generate bias-corrected and accelerated bootstrap confidence intervals. These intervals were calculated for the mean differences in clinical outcomes, specifically low-density lipoprotein cholesterol (LDL-C) levels and the number of cardiovascular-related hospitalizations, as well as the mean differences in annual direct medical costs between patients treated with PCSK9 inhibitors (such as evolocumab and alirocumab) and those receiving statins and ezetimibe [36]. The LDL-C levels were expressed in millimoles per liter (mmol/L), while medical costs were expressed in United States Dollars (USD) using the standard conversion rate of 3.75 Saudi Riyals per 1 USD. All statistical analyses were conducted using SAS^®^ version 9.4 (SAS Institute, Cary, NC, USA).

### 2.3. Sensitivity Analysis

To examine the impact of changing the acquisition prices of PCSK9 inhibitors (evolocumab and alirocumab), a one-way sensitivity analysis was conducted, considering three different scenarios. The procurement of pharmaceuticals for the public health sector is managed through a national procurement company, also known as NUPCO. The first scenario included the public acquisition prices of evolocumab and alirocumab from the SFDA website, which are USD 234.46 for each pre-filled pen of 140 mg/mL or USD 468.92 for 420 mg solution for injection in the cartridge for evolocumab, and USD 249.75 for each pre-filled pen of alirocumab of 150 mg/mL or 75 mg/mL. The second scenario included discounted prices of USD 135.79 for each pre-filled pen of 140 mg/mL or USD 412.80 for the 420 mg solution for injection in the cartridge for evolocumab, and USD 96.32 for each pre-filled pen of alirocumab at 150 mg/mL or 75 mg/mL. The third scenario included discounted prices of USD 100.43 for each pre-filled pen of 140 mg/mL or USD 301.30 for 420 mg solution for injection in a cartridge for evolocumab, and USD 96.32 for each pre-filled pen of alirocumab of 150 mg/mL or 75 mg/mL. These discounted prices in scenarios two and three were obtained from five medical supply chain specialists working in different public health institutions.

## 3. Results

### 3.1. Patients’ Baseline Characteristics

**Figure 1:**
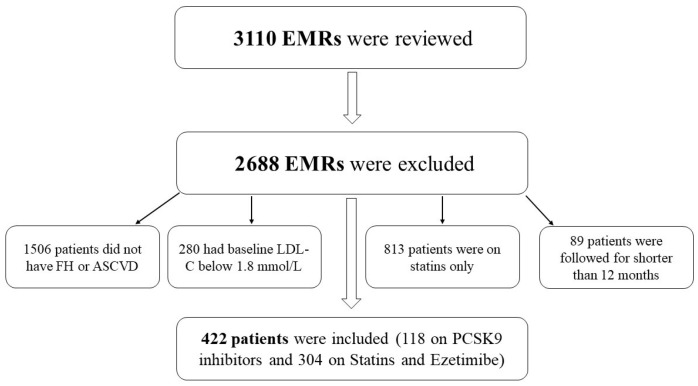
Schematic diagram of patients’ recruitment.

Out of 3110 medical files that were reviewed, 2688 patients were excluded, as shown in Figure 1. The number of patients who met the inclusion criteria and were included in the analysis was 422, of whom 118 were on PCSK9 inhibitors and 304 were on statins plus ezetimibe. The majority of patients on PCKS9 inhibitors (90.68%) were taking evolocumab, and only 11 patients (9.32%) were on alirocumab. Patients on PCSK9 inhibitors were younger than their counterparts on statins plus ezetimibe (51 years vs. 68 years, *p*-value < 0.001). Most patients were males (61.85%), with an average duration of illness of approximately five years. Moreover, the mean body mass index (BMI) was approximately 30, with no significant difference between patients treated with PCSK9 inhibitors and their counterparts on statins plus ezetimibe (29.59 vs. 30.59, *p*-value = 0.1090). Patients treated with statins plus ezetimibe were, on average, treated for a more extended period compared to their counterparts on PCSK9 inhibitors (5.39 years vs. 3.03 years, *p*-value < 0.001). Additionally, the percentage of patients treated with statins plus ezetimibe who had other comorbidities was higher compared to their counterparts on PCSK9 inhibitors (*p*-value < 0.05). For example, approximately 70% of patients treated with statins plus ezetimibe had diabetes, 20% had chronic kidney disease (CKD), 53% had hypertension, 24% had ST-elevation myocardial infarction (STEMI), and 51% had Non-STEMI elevation myocardial infarction (NSTEMI) compared to about 43%, 4%, 28%, 12%, and 24%, respectively, among their counterparts on PCSK9 inhibitors. In contrast, 19.5% of patients taking PCSK9 inhibitors reported being smokers, whereas only 0.33% of patients on statins combined with ezetimibe did so (*p*-value < 0.001). Finally, the baseline LDL-C level for patients on PCSK9 inhibitors was 5.19 mmol/L compared to 3.35 mmol/L among their counterparts on statins plus ezetimibe (*p*-value < 0.001); the mean levels of other lipid profile measures (HDL, triglycerides, and total cholesterol) were also higher among patients on PCSK9 inhibitors compared to their counterparts on statins plus ezetimibe as shown in Table 1.

### 3.2. The Effects of PCSK9 Inhibitors and the Combination of Statins with Ezetimibe on Lipid Profile

The mean lipid profile measures levels at baseline and follow-up, and the mean differences are shown in Table 2. The mean baseline LDL-C level for patients on PCSK9 inhibitors was 1.43 mmol/L (95% CI: 0.964–1.899 mmol/L), higher than their mean follow-up level (*p*-value < 0.001). Likewise, the baseline LDL-C level for patients on statins plus ezetimibe was 0.644 mmol/L (95% CI: 0.464–0.823 mmol/L), higher than their mean follow-up level (*p*-value < 0.0001). The mean baseline triglyceride levels for patients on PCSK9 inhibitors were 0.307 mmol/L (95% CI: −0.031–0.645 mmol/L) higher than their mean follow-up level. However, this difference was not statistically significant (*p*-value = 0.074). Similarly, the baseline triglyceride level for patients on statins plus ezetimibe was 0.168 mmol/L (95% CI: 0.049–0.287 mmol/L) higher than their mean follow-up level (*p*-value = 0.005). Interestingly, patients on statins plus ezetimibe had slightly (−0.022 mmol/L) but significantly lower HDL-C levels (*p*-value = 0.0436) at follow-up. Finally, the mean reduction in total cholesterol level was greater for patients on PCSK9 inhibitors compared to those on statins plus ezetimibe (1.642 vs. 0.684 mmol/L, *p*-value < 0.001).

### 3.3. Medical Costs Associated with Treating Hypercholesterolemia with PCSK9 Inhibitors Versus Statins Plus Ezetimibe

**Figure 2:**
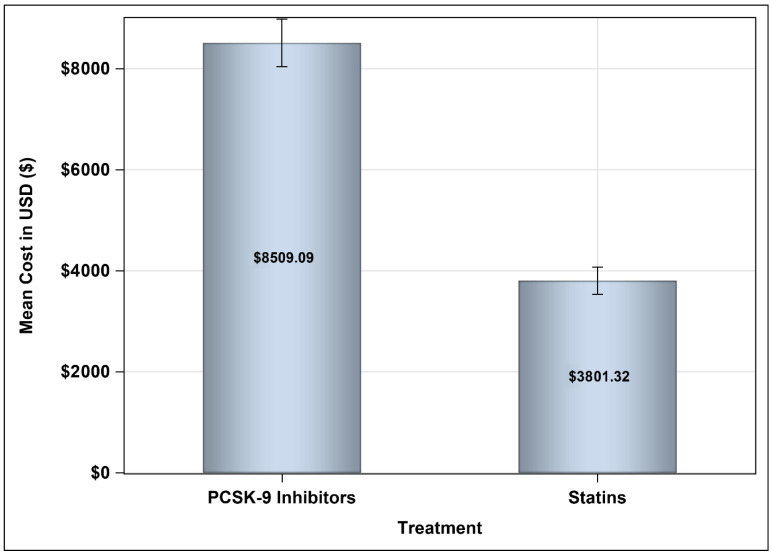
Mean annual medication cost for patients on PCSK9 inhibitors and statins based on the SFDA prices.

**Figure 3:**
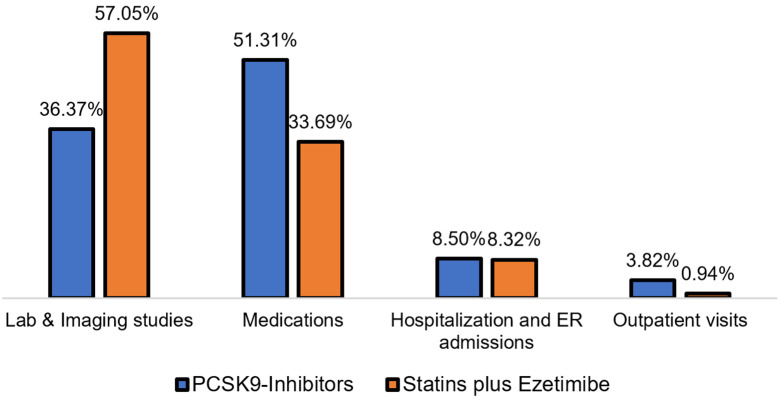
Average percentages of each medical cost category among patients on PCSK9 inhibitors and statins plus ezetimibe.

The mean annual medication costs for patients treated with PCSK9 inhibitors were USD 8509.09 versus USD 3801.32, resulting in a mean difference (MD) of USD 4707.76 ([95% CI: 4187.9–5227.6], *p*-value = <0.001), using the SFDA prices for prescription drugs, as shown in Figure 2. Moreover, the cost of prescription medications for patients treated with PCSK9 inhibitors accounted for approximately 51% of the total annual direct medical costs, compared to 33.69% among their counterparts, as shown in Figure 3.

### 3.4. Cost-Effectiveness of PCSK9 Inhibitors Versus Statins Plus Ezetimibe for LDL-C Level Reduction

**Figure 4:**
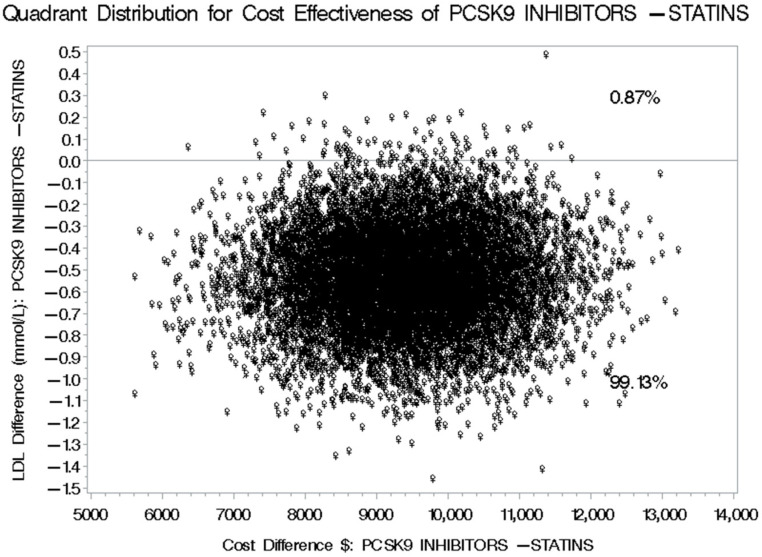
Bootstrap Distribution of Cost-Effectiveness: Comparative Analysis between PCSK9 inhibitors and Statins plus Ezetimibe for their impact on LDL-C levels based on SFDA Prices of Prescription Drugs.

The mean annual direct medical cost for patients treated with PCSK9 inhibitors was USD 16,466.78 before adjustment and USD 19,061.95 after adjustment using the SFDA prices for prescription medications, compared to USD 12,510.40 before adjustment and USD 11,503.07 after adjustment among their counterparts treated with statins plus ezetimibe. This has resulted in a mean difference in the direct annual medical costs of USD 3956.38 and USD 7558.88 between patients treated with PCSK9 inhibitors and their counterparts on statins plus ezetimibe, using the unadjusted and adjusted mean annual direct medical costs, respectively. Alternatively, the mean difference in the LDL-C reduction between the patients treated with PCSK9 inhibitors and their counterparts treated with statins plus ezetimibe was −0.79 mmol/L [95% CI: −1.46–−0.60], which means that the use of PCSK9 inhibitor for the management of hypercholesterolemia will results in an incremental cost of USD 9618.98 [95% CI: 5021.47 to 19,182.76] for each additional one mmol/L reduction in LDL-C level compared to statins plus ezetimibe (Table 3). Moreover, two cost-effectiveness quadrants were generated in this first scenario (SFDA prices), and the management of hypercholesterolemia using PCSK9 inhibitors was associated with higher costs but more significant reductions in LDL-C levels 99.13% of the time. In comparison, higher costs with smaller reductions in LDL-C were observed 0.87% of the time, as shown in Figure 4.

**Figure 5:**
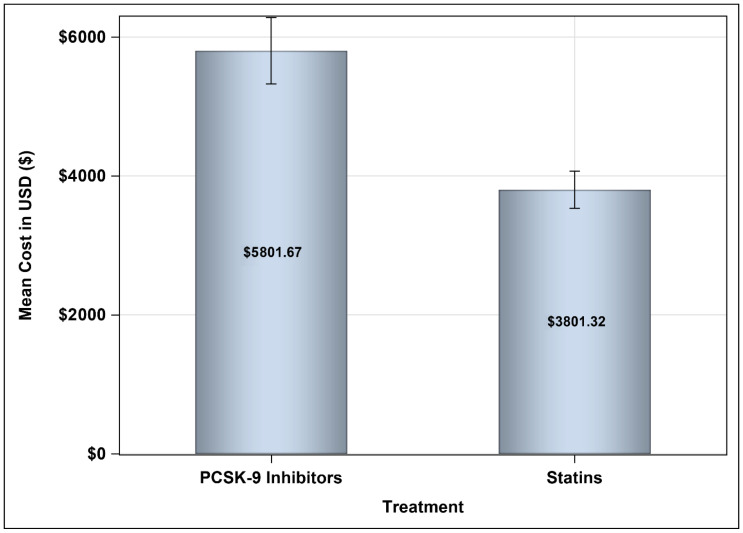
Mean annual medication costs for patients on PCSK9 inhibitors and statins based on the discounted prescription medication prices for public health institutions for the years 2021–2023.

**Figure 6:**
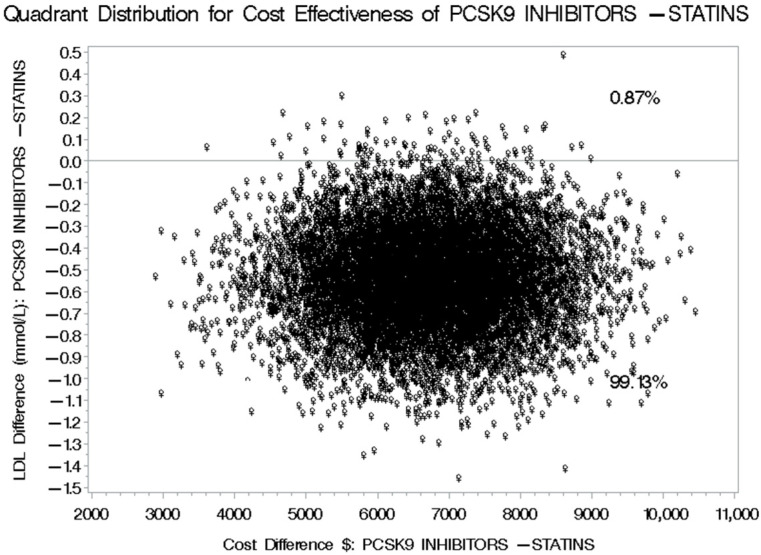
Bootstrap Distribution of Cost-Effectiveness: Comparative Analysis between PCSK9 inhibitors and Statins plus Ezetimibe for their impact on LDL-C levels based on the discounted prescription medication prices for public health institutions for the years 2021–2023.

**Figure 7:**
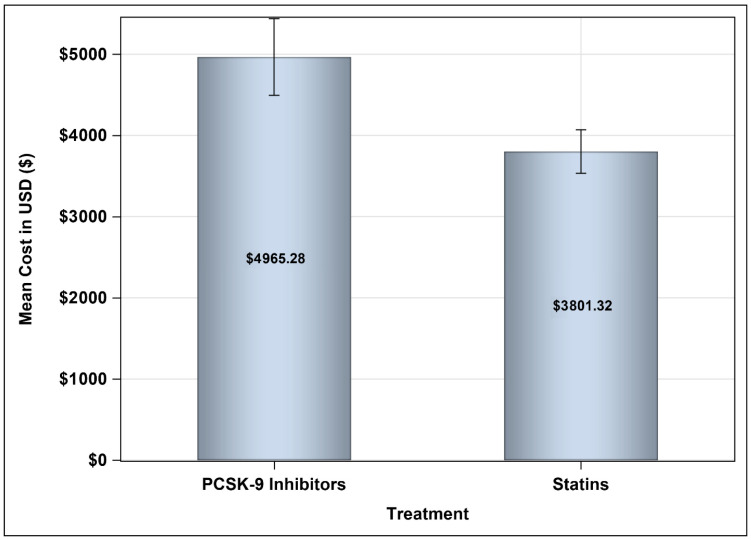
Mean annual medication costs for patients on PCSK9 inhibitors and statins based on the discounted prescription medication prices for public health institutions for the years 2024–2026.

**Figure 8:**
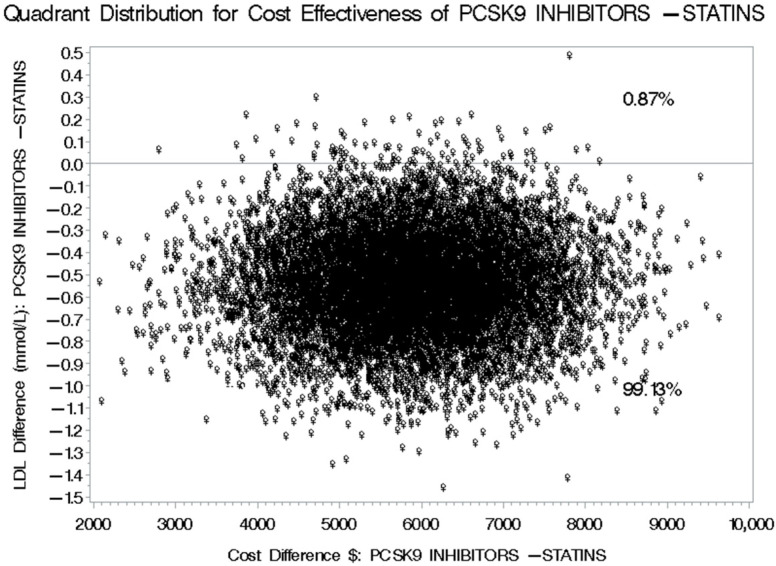
Bootstrap Distribution of Cost-Effectiveness: Comparative Analysis between PCSK9 inhibitors and Statins plus Ezetimibe for their impact on LDL-C levels based on the discounted prescription medication prices for public health institutions for the years of 2024–2026.

The second scenario included the discounted prices of PCSK9 inhibitors for public health institutions for the 2021–2023 tender, as shown in Figure 5. The difference in the mean annual medication costs between the between patients on PCSK9 inhibitors and their counterparts on statins plus ezetimibe was USD 2000.4 ([95% CI: 1478.4 to 2522.3], *p*-value < 0.0001), which has resulted in a mean difference in the annual direct medical costs between patients on PCSK9 inhibitors and their counterparts on statins plus ezetimibe of USD 4875.14 (95% CI: 4623.54 to 8769.09) as shown in Table 4, which led to a lower incremental cost-effectiveness ratio (ICER) of USD 6171.06 [95% CI: 3166.80 to 14,615.15] compared to USD 9618.98 [95% CI: 5021.47 to 19,182.76] in the first scenario (~36% reduction) for each additional one mmol/L decrease in LDL-C. The bootstrap cost-effectiveness distributions resulted in two quadrants, similar to the first scenario, where PCSK9 inhibitors resulted in higher costs and lower LDL-C levels 99.13% of the time, and higher costs and smaller benefits for the management of hypercholesterolemia in 0.87% of the time, compared to statins plus ezetimibe, as shown in Figure 6. Alternatively, the third scenario, which included discounted prices for PCSK9 inhibitors prices (mean annual medication cost of USD 4965.28) for public health institutions for the years 2024–2026 tender with a mean difference (MD) between patients on PCSK9 inhibitors and their counterparts on statins plus ezetimibe of USD 1164.0 ([95% CI: 643.9 to 1684.1], *p*-value < 0.001), as shown in Figure 7, resulted in an ICER of USD 5093.04 [95% CI: 2593.69 to 13,246.51] based on the difference in the mean annual total direct medical costs between patients treated with PCSK9 inhibitors compared to their counterparts on statins plus ezetimibe as shown in Table 5, and similar bootstrap cost effectiveness quadrants as shown in Figure 8.

### 3.5. Cost-Effectiveness of PCSK9 Inhibitors Versus Statins Plus Ezetimibe for Reducing Cardiovascular-Related Hospitalization

**Figure 9:**
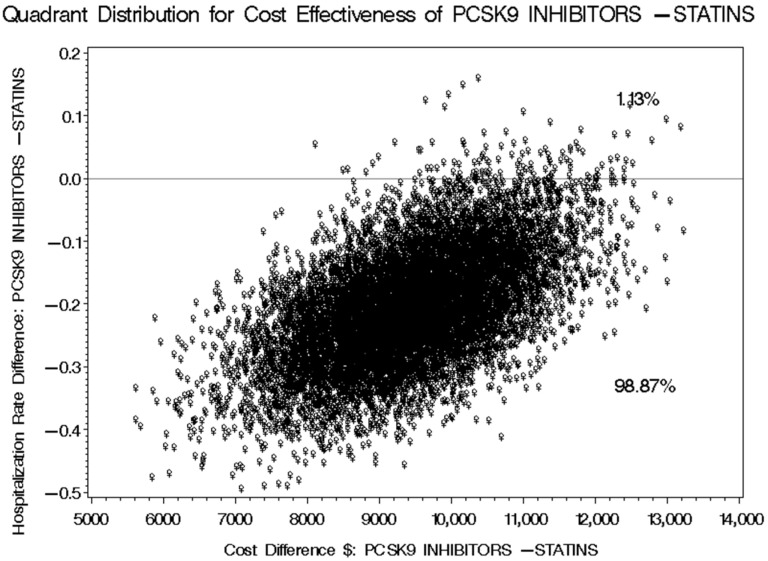
Bootstrap Distribution of Cost-Effectiveness: Comparative Analysis between PCSK9 inhibitors and Statins plus Ezetimibe for their impact on the number of cardiovascular-related hospitalizations based on SFDA Prices of Prescription Drugs.

**Figure 10:**
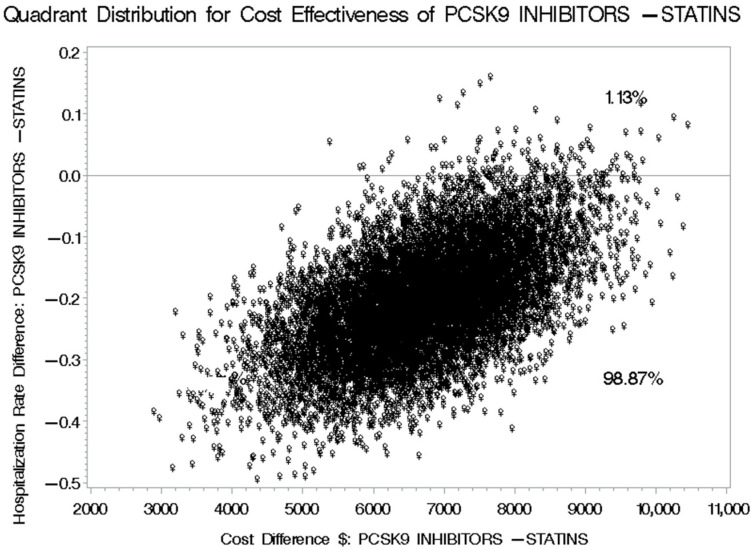
Bootstrap Distribution of Cost-Effectiveness: Comparative Analysis between PCSK9 inhibitors and Statins plus Ezetimibe for their impact on the number of cardiovascular-related hospitalizations based on the discounted prescription medication prices for public health institutions for the years 2021–2023.

**Figure 11:**
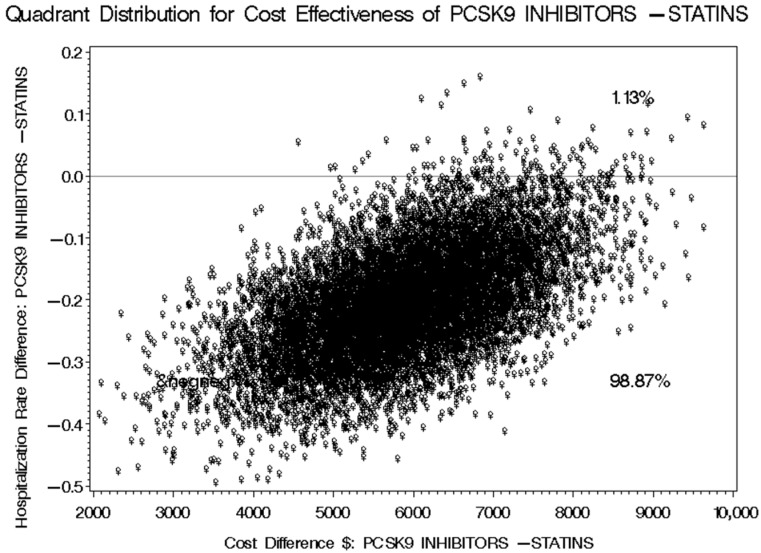
Bootstrap Distribution of Cost-Effectiveness: Comparative Analysis between PCSK9 inhibitors and Statins plus Ezetimibe for their impact on the number of cardiovascular-related hospitalizations based on the discounted prescription medication prices for public health institutions for the years 2024–2026.

The mean number of cardiovascular-related hospitalizations for patients receiving PCSK9 inhibitors was 0.645, compared to 0.808 for those on statins plus ezetimibe. This results in a difference of −0.163 (95% CI: −0.287 to −0.0601) in favor of PCSK9 inhibitors, as detailed in Table 6. This variation in cardiovascular-related hospitalizations translates to an incremental cost-effectiveness ratio (ICER) of USD 46,374.23 (95% CI: 25,544.77 to 191,508.48), indicating the additional cost incurred for each avoided cardiovascular-related hospitalization when using PCSK9 inhibitors for managing hypercholesterolemia instead of statins plus ezetimibe, based on the prices set by the SFDA for prescription medications. Utilizing the discounted prices of PCSK9 inhibitors in public health institutions for the second (tender prices 2021–2023) and third (tender prices 2024–2026) scenarios would yield ICERs of USD 29,908.83 (95% CI: 16,109.89 to 145,908.31) and USD 24,684.05 (95% CI: 13,194.42 to 132,244.75), respectively, as presented in Table 7 and Table 8. The bootstrap cost-effectiveness distributions for these three scenarios generated two distinct quadrants. When comparing PCSK9 inhibitors to statins plus ezetimibe, the outcomes indicated that there would be higher costs and a greater number of cardiovascular-related hospitalizations 1.13% of the time, while 98.87% of the time, there would be higher costs and a reduced number of cardiovascular-related hospitalizations, as illustrated in Figure 9, Figure 10 and Figure 11.

## 4. Discussion

Patients diagnosed with hypercholesterolemia face an elevated risk of cardiovascular disease (CVD), which stands as the foremost cause of mortality worldwide. In Saudi Arabia, CVD accounts for over one-third of total deaths, underscoring the severity of this public health challenge [2,4,5,8]. Hence, the administration of lipid-lowering medications is essential to effectively manage hypercholesterolemia and significantly reduce the likelihood of major adverse cardiovascular events (MACE) [10]. Among the various classes of lipid-lowering drugs, PCSK9 inhibitors, such as evolocumab and alirocumab, have shown remarkable efficacy in reducing low-density lipoprotein cholesterol (LDL-C) levels in numerous clinical trials. However, a comprehensive evaluation of their cost-effectiveness using real-world data across diverse healthcare settings has yet to be thoroughly investigated [13]. In this particular study, it was revealed that PCSK9 inhibitors yield more effective LDL-C reductions compared to the traditional regimen of statins combined with ezetimibe. On average, incorporating PCSK9 inhibitors into treatment protocols led to a more substantial LDL-C reduction of 0.79 mmol/L (equivalent to 30.55 mg/dL) over what was achieved with statins plus ezetimibe. When comparing the two treatment approaches, PCSK9 inhibitors (namely evolocumab and alirocumab) resulted in a mean decrease in LDL-C levels by 15.22% [95% CI: (11.56 to 28.13), *p*-value < 0.0001], based on baseline LDL-C measurements. In contrast, prior meta-analyses, such as one conducted by Zhang et al., have demonstrated a more pronounced 46.86% reduction in LDL-C levels [95% CI: (37.72 to 54.99)] across pooled data from 14 clinical trials [20]. Additionally, real-world evidence from Taiwan suggested even greater reductions at 49.6% [27]. This variability in effectiveness highlights the diverse impacts of PCSK9 inhibitors on LDL-C reduction among different patient populations [20,27]. Despite their effectiveness, the higher associated medical expenses of using PCSK9 inhibitors compared to the standard care (statins plus ezetimibe) led to an incremental cost-effectiveness ratio (ICER) of USD 9618.98 for every 1 mmol/L decrease in LDL-C levels, calculated based on prices set by the SFDA. While some studies have recognized a positive correlation between LDL-C reductions and health-related quality of life (HRQoL), notably associating a 30% decrease in LDL-C levels with a 0.9 quality-adjusted life year (QALY) gain, this study could not establish similar findings using the available data [37]. Furthermore, achieving a 30% reduction in baseline LDL-C levels (approximately 1.557 mmol/L) was linked to an incremental cost of USD 14,976.75, reflecting the economic implications of such treatment protocols as uncovered in this study. Other cost-effectiveness analyses examining PCSK9 inhibitors have produced mixed results, particularly those that presumed more significant LDL-C reductions based on evidence from randomized clinical trials [26]. For instance, in a cost-effectiveness evaluation focusing on patients with FH and triple-vessel coronary artery disease (CAD), overall utilization of PCSK9 inhibitors was deemed not cost-effective; however, the findings suggested potential cost-effectiveness for those with poorly controlled FH [28]. Additionally, a study performed within the framework of the National Health Service (NHS) in the United Kingdom determined that the application of either evolocumab or alirocumab exhibited negligible probabilities of being cost-effective at the NHS’s cost-effectiveness threshold (CET) of £30,000 per QALY for secondary cardiovascular prevention [29]. While evolocumab was reportedly considered cost-effective for patients with clinically evident atherosclerotic cardiovascular disease (ASCVD) and individuals with heterozygous FH in the Saudi context, an analysis primarily informed by data from a single clinical trial, the research did not account for the latest CET published for Saudi Arabia, which narrows the drug’s cost-effectiveness to patients with heterozygous FH only [33]. Moreover, the ICERs mentioned in Alghamdi et al. could vary significantly if the efficacy estimates from this study were integrated into their model [33].

The mean difference regarding cardiovascular-related hospitalizations between patients receiving PCSK9 inhibitors and those on statins combined with ezetimibe was notably lower in the PCSK9 inhibitor group, with a mean difference of −0.163 [95% CI: (– 0.287 to −0.0601)]. The associated incremental costs ranged from USD 24,684.05 to USD 46,374.23 for each cardiovascular-related hospitalization that was averted. Furthermore, another investigation into the effects of evolocumab on cardiovascular-related mortality—utilizing data from the FOURIER cardiovascular outcomes trial and contrasting it with mortality figures from the clinical study report (CSR) provided by the manufacturer—indicated that patients treated with evolocumab experienced a higher rate of cardiovascular-related deaths in comparison to those receiving a placebo [38]. Given these findings, it is imperative to devise effective market access agreements that link the reimbursement of PCSK9 inhibitors to clinical outcomes to alleviate the uncertainties surrounding the efficacy of these therapeutic agents. Furthermore, a thorough examination of the ramifications of current agreements related to PCSK9 inhibitors on the cost-effectiveness of healthcare spending within Saudi Arabia is warranted [39].

### Limitations of the Study

This study represents the first multi-center investigation into the clinical and economic implications of utilizing PCSK9 inhibitors in the management of hypercholesterolemia within public healthcare frameworks in Saudi Arabia, leveraging real-world data. However, several limitations must be carefully considered to provide a balanced understanding of the findings. Firstly, the study did not confirm the diagnosis of heterozygous familial hypercholesterolemia (FH) through genetic testing, nor was this diagnosis reliably documented in the electronic medical records (EMRs) for patients receiving PCSK9 inhibitors or those treated with a combination of statins and ezetimibe. As a result, the analysis did not stratify the cohorts according to the treatment modalities, which would have allowed for a more nuanced examination of the effectiveness and associated costs of PCSK9 inhibitors compared to conventional therapies. Moreover, the possibility of information bias cannot be entirely dismissed, as the data collected were sourced from EMRs that may not capture the full clinical picture. Additionally, the absence of a cost-effectiveness analysis that incorporates QALYs is a significant shortcoming in the study, primarily due to the lack of data collected on health-related quality of life (HRQoL) among participants. This limits the ability to assess the long-term value of these therapies comprehensively. Furthermore, while the study attempted to adjust for differences in patient characteristics through inverse probability of treatment weighting, residual confounding remains a concern, particularly as the cohort receiving statins and ezetimibe was older and presented with a higher prevalence of comorbid conditions. This factor could skew the results and affect the interpretation of the comparative effectiveness of the treatments. Another important variable that was not evaluated is medication adherence. Although the study noted that patients consistently refilled their prescriptions, adherence to PCSK9 inhibitors, which require regular injections, is critical for achieving therapeutic effectiveness. Previous research, including a recent publication from a Chinese context, indicates that many patients may struggle with persistence on injectable therapies [40]. Despite this, it is worth mentioning that all patients in this study displayed a history of uninterrupted prescription refills. Interestingly, adherence and persistence rates among those on PCSK9 inhibitors were reported to be higher than those for patients on oral lipid-lowering agents like ezetimibe, based on findings from an observational study utilizing pharmacy fill claims data from the United States [41]. Lastly, it is crucial to note that this study was conducted exclusively within tertiary medical centers located in Riyadh, which may limit the generalizability of its findings. The exclusion of data from other regions in Saudi Arabia could potentially overlook variations in healthcare practices and patient populations across the country. Despite the aforementioned limitations, this study represents an effort that offers valuable insights into the clinical and economic evaluation of PCSK9 inhibitors in Saudi Arabia, a nation grappling with high rates of dyslipidemia and cardiovascular disease [6,9]. The increasing reliance on these therapies imposes substantial financial demands on the healthcare budget, making this study particularly relevant in the context of ongoing healthcare planning and resource allocation.

## 5. Conclusions

The advent of PCSK9 inhibitors has transformed the management of hypercholesterolemia, especially for patients who do not achieve adequate control through traditional therapies. These agents significantly reduce low-density lipoprotein cholesterol (LDL-C) levels and decrease cardiovascular-related hospitalizations compared to conventional treatments, such as statins and ezetimibe. However, their high cost poses challenges for widespread adoption, raising concerns about the accessibility and sustainability of these therapies. The findings of this study suggest that the reduction in LDL-C may not be as pronounced as previously reported, necessitating careful interpretation of the data and further investigation. Future research should focus on more robust study designs to evaluate the cost-effectiveness of PCSK9 inhibitors in managing hypercholesterolemia in Saudi Arabia, considering both clinical outcomes and economic implications, and the broader impact on healthcare systems and patient quality of life.

## Figures and Tables

**Table 1:** Patient baseline characteristics.

Characteristic	PCSK9 Inhibitors (N = 118)	Statins Plus Ezetimibe (N = 304)	*p*-Value	Total
Age (yrs.), mean ± SD	50.96 ± 14.49	67.72 ± 13.79	<0.001	63.04 ± 15.88
**Gender, n(%)**				
Male	79 (66.95)	182 (59.87)	0.179	261 (61.85)
Female	39 (33.05)	122 (40.13)		161 (38.15)
Duration of illness (yrs.), mean ± SD	4.89 ± 2.69	5.39 ± 1.98	0.068	5.25 ± 2.22
Body Mass Index (BMI), mean ± SD	29.59 ± 5.92	30.59 ± 5.69	0.109	30.31 ± 2.69
Duration of therapy (yrs.), mean ± SD	3.03 ± 1.38	5.39 ± 1.98	<0.001	4.73 ± 2.12
**Comorbidities, n (%)**				
Chronic Kidney Disease (CKD)	5 (4.24)	61 (20.07)	<0.001	66 (15.64)
Diabetes	51 (43.22)	214 (70.39)	<0.001	265 (62.80)
Hypertension	33 (27.97)	161 (52.96)	<0.001	194 (45.97)
ST Elevation Myocardial Infarction (STEMI)	14 (11.86)	72 (23.68)	0.006	86 (20.38)
Non-ST-elevation myocardial infarction (NSTEMI)	28 (23.73)	156 (51.32)	<0.001	184 (43.60)
**Smoking status**				
Smoker	23 (19.49)	1 (0.33)	<0.001	24 (5.69)
Ex-smoker	6 (5.08)	0 (0.0)		6 (1.42)
Non-smoker	89 (75.42)	303 (99.67)		392 (92.89)
**Lab values**				
LDL (mmol/L), mean ± SD	5.19 ± 3.62	3.35 ± 1.89	<0.001	3.87 ± 2.63
Triglyceride (mmol/L), mean ± SD	2.04 ± 2.29	1.81 ± 1.87	0.328	1.87 ± 1.99
HDL (mmol/L), mean ± SD	1.07 ± 0.29	0.99 ± 0.26	0.012	1.02 ± 0.27
Total cholesterol (mmol/L), mean ± SD	7.12 ± 3.74	4.96 ± 1.94	<0.001	5.56 ± 2.75

**Table 2:** Difference in lipid panel levels between baseline and follow-up.

Variable	Mean at Baseline (mmol/L) ± SD	Mean at Follow-Up (mmol/L) ± SD	Mean Difference (mmol/L) (95% Confidence Interval)	*p*-Value
LDL for PCSK9 inhibitors	5.19 ± 3.62	3.76 ± 3.16	1.432 (0.964–1.899)	<0.001
LDL for statins plus ezetimibe	3.35 ± 1.89	2.71 ± 1.45	0.644 (0.464–0.823)	<0.001
Triglyceride for PCSK9 inhibitors	2.04 ± 2.29	1.74 ± 1.04	0.307 (−0.031–0.645)	0.074
Triglyceride for statins plus ezetimibe	1.81 ± 1.87	1.64 ± 1.27	0.168 (0.049–0.287)	0.005
HDL for PCSK9 inhibitors	1.07 ± 0.29	1.07 ± 0.28	−0.002 (−0.035–0.030)	0.886
HDL for statins plus ezetimibe	0.99 ± 0.26	0.97 ± 0.189	0.022 (0.0006–0.0433)	0.043
Total cholesterol for PCSK9 inhibitors	7.12 ± 3.74	5.48 ± 3.29	1.642 (1.156–2.129)	<0.001
Total cholesterol for statins plus ezetimibe	4.96 ± 1.94	4.27 ± 1.54	0.684 (0.497–0.872)	<0.001

**Table 3:** The mean LDL (mmol/L) level reduction and treatment cost for patients on PCSK9 inhibitors (N = 118) versus statins and ezetimibe (N = 304) using the SFDA Prices of Prescription Drugs.

Variable	PCSK9 Inhibitors	Statins Plus Ezetimibe	Mean Difference (95% Confidence Interval)
Cost of treatment (USD), mean ± SD	19,061.95 ± 10,254.45	11,503.07 ± 12,834.15	7559 (7331.35–11,509.66)
Difference in LDL (mmol/L)	−1.43 ± 2.57	−0.64 ± 1.59	−0.79 (−1.46–−0.60)

**Table 4:** The mean LDL (mmol/L) level reduction and treatment cost for patients on PCSK9 inhibitors (N = 118) versus statins and ezetimibe (N = 304) using the discounted prescription medication prices for public health institutions for the years 2021–2023.

Variable	PCSK9 Inhibitors	Statins Plus Ezetimibe	Mean Difference (95% Confidence Interval)
Cost of treatment (USD), mean ± SD	16,371.59 ± 10,277.46	11,496.45 ± 12,834.15	4875.14 (4623.54–8769.09)
Difference in LDL (mmol/L)	−1.43 ± 2.57	−0.64 ± 1.59	−0.79 (−1.46–−0.60)

**Table 5:** The mean LDL (mmol/L) level reduction and treatment cost for patients on PCSK9 inhibitors (N = 118) versus statins and ezetimibe (N = 304) using the discounted prescription medication prices for public health institutions for the years 2024–2026.

Variable	PCSK9 Inhibitors	Statins Plus Ezetimibe	Mean Difference (95% Confidence Interval)
Cost of treatment (USD), mean ± SD	15,524.19 ± 10,260.67	11,500.72 ± 12,834.15	4023.5 (3786.80–7947.91)
Difference in LDL (mmol/L)	−1.43 ± 2.57	−0.64 ± 1.59	−0.79 (−1.46–−0.60)

**Table 6:** The mean hospitalization number and treatment cost for patients on PCSK9 inhibitors (N = 118) versus statins and ezetimibe (N = 304) using the SFDA Prices of Prescription Drugs.

Variable	PCSK9 Inhibitors	Statins Plus Ezetimibe	Mean Difference (95% Confidence Interval)
Cost of treatment (USD), mean ± SD	19,061.95 ± 10,254.45	11,503.07 ± 12,834.15	7559 (7331.35–11,509.66)
Mean number of cardiovascular-related hospitalizations	0.645 ± 0.93	0.808 ± 0.82	−0.163 (−0.287–−0.0601)

**Table 7:** The mean hospitalization number and treatment cost for patients on PCSK9 inhibitors (N = 118) versus statins and ezetimibe (N = 304) using the discounted prescription medication prices for public health institutions for the years 2021–2023.

Variable	PCSK9 Inhibitors	Statins Plus Ezetimibe	Mean Difference (95% Confidence Interval)
Cost of treatment (USD), mean ± SD	16,371.59 ± 10,277.46	11,496.45 ± 12,834.15	4875.14 (4623.54–8769.09)
Mean number of cardiovascular-related hospitalizations	0.645 ± 0.93	0.808 ± 0.82	−0.163 (−0.287–0.0601)

**Table 8:** The mean hospitalization number and treatment cost for patients on PCSK9 inhibitors (N = 118) versus statins and ezetimibe (N = 304) using the discounted prescription medication prices for public health institutions for the years 2024–2026.

Variable	PCSK9 Inhibitors	Statins Plus Ezetimibe	Mean Difference (95% Confidence Interval)
Cost of treatment (USD), mean ± SD	15,524.19 ± 10,260.67	11,500.72 ± 12,834.15	4023.5 (3786.80–7947.91)
Mean number of cardiovascular-related hospitalizations	0.645 ± 0.93	0.808 ± 0.82	−0.163 (−0.287–0.0601)

## Data Availability

The original contributions presented in this study are included in the article. Further inquiries can be directed to the corresponding author.

## Abbreviations

CVD	Cardiovascular disease
MACE	Major Adverse Cardiovascular Events
FH	Familial Hypercholesterolemia
PCSK9	Proprotein Convertase Subtilisin/Kexin type 9
ANGPTL3	Angiopoietin-like 3
ASCVD	Atherosclerotic Cardiovascular Disease
NNTs	Numbers Needed to Treat
EMRs	Electronic Medical Records
CKD	Chronic Kidney Disease
SFDA	Saudi Food and Drug Authority
HRQoL	Health-Related Quality of Life
mAbs	Monoclonal antibodies
USD	United States Dollars
QALY	Quality-adjusted life years
CSR	Clinical Study Report
NHS	National Health Service
CET	Cost-Effectiveness Threshold
ICER	Incremental Cost-Effectiveness Ratio
